# Asymptomatic Cervical Amputation Caused by Uterine Torsion in a Non-Gravid Woman

**DOI:** 10.3390/jcm13237356

**Published:** 2024-12-03

**Authors:** Milan Stefanović, Predrag Vukomanović, Ranko Kutlešić, Milan Trenkić, Vanja Dimitrov, Aleksa Stefanović

**Affiliations:** 1 Faculty of Medicine, University of Niš, Blvd. Dr Zorana Đinđića 81, 18000 Niš, Serbia; predragvukomanovic@yahoo.com (P.V.); kutlesicr@medfak.ni.ac.rs (R.K.); trenkic@gmail.com (M.T.); vanjadimitrov@gmail.com (V.D.); lexstef97@gmail.com (A.S.); 2 Gynecolocgy and Obstetrics Clinic, Clinical Center Niš, Blvd. Dr Zorana Đinđića 48, 18000 Niš, Serbia

**Keywords:** torque, uterine anomalies, uterine torsion, post-menopause, acute abdomen

## Abstract

**Background:** Uterine torsion represents a rare condition that may occur during pregnancy or in non-gravid women. This condition is difficult to diagnose, since there are no specific signs besides abdominal pain. Thus, most of the cases are not diagnosed correctly before a surgical procedure and may result in complications and poor outcomes. **Methods**: We present the first case of uterine torsion of 1080 degrees counterclockwise, with asymptomatic cervical amputation. **Results:** The intraoperative finding was a uterus that was twisted 1080 degrees around its longitudinal axis, a large fibroid > 15 cm, a large ovarian tumor > 15 cm, and a missing cervix. Upon further inspection, the cervix was found, completely separated from the body of the uterus. After the surgery, the patient remained stable, and her postoperative course was uneventful. She was discharged on the eighth postoperative day. No complications were detected 2 months after the surgery. **Conclusions:** This specific case is extremely unique, being the only one in searched literature with a 1080° torsion and an amputated cervix. Uterine torsion, especially in postmenopausal women, is a highly rare condition and is difficult to diagnose, with potentially serious outcomes. Any doubts should be assessed as quickly as possible and be dealt with appropriately. If possible, MRI and CT scans could be of great help in differential diagnosis.

## 1. Introduction

Torsion of female reproductive organs typically causes sudden, severe pain that does not respond to medication and may present a gynecological and/or obstetric emergency. Pain of differing intensity is usually the main symptom in these cases; however, patients may also develop complete hemodynamic shock [1,2]. When considering female reproductive organ torsions, ovarian torsions have been relatively frequently reported on, since they occur in 5.9 per 100,000 women [3] and make up around 3% of all surgical emergencies [4]. Torsions of pedunculated fibroids are less frequent, with only about 0.25% of surgical cases being reported [5].

Uterine torsion is an extremely rare phenomenon, occurring mostly in pregnant women, and is an even rarer phenomenon in non-pregnant women, with just more than 250 reported cases of uterine torsions in non-pregnant women [6,7] and 14 documented cases in postmenopausal women in the 20 years prior to 2021 [8].

Torsion of the uterus is defined as a rotation of the uterus around its longitudinal axis by more than 45 degrees [9]. According to the majority of the reported cases, the torsion is mostly clockwise (dextrorotatory), occurs in 2/3 of cases [9,10], and usually ranges from 45 to 180 degrees [11]. It should be pointed out that uterine torsions up to 720 degrees had been reported [12,13,14]. Abdominal pain (with differing intensity) represents the most common symptom present in gravid or non-gravid women with uterine torsion, as stated earlier. However, this symptom is not enough to provide an early diagnosis of uterine torsion and prevent possible complications. The lack of specific symptoms that are caused by uterine torsion may lead to delayed or incorrect diagnosis, which, in the end, may result in serious complications, especially in the younger population with a possibility to preserve fertility. The delay of uterine torsion diagnosis for a longer period may lead to reduced blood flow to the organ and could result in uterine rupture or gangrenous development, which would inevitably end with a total hysterectomy and fertility loss. The presented case describes a uterus torsion of 1080° around its longitudinal axis, together with an ovarian tumor, leiomyoma, and a missing cervix in a postmenopausal woman. To the best of our knowledge, this is the first report of a cervix that is completely separated from the body of the uterus, caused by the torsion of the uterus.

## 2. Case Presentation

A 47-year-old woman was admitted to the hospital with mild intensity pain located in the lower abdomen. The pain had been present for the past month, as described by the patient. She had been continuously on pain control medication during that month and had not visited her gynecologist. While reviewing her medical history, we discovered the presence of an untreated large uterine leiomyoma, which was diagnosed a couple of years ago. Even with the presence of a growing mass in her abdomen, she hardly experienced any symptoms and had been mostly asymptomatic until her admission to the hospital and in the month leading to it.

Upon admission to the hospital, the patient had normal vital signs and was in a stable state. A clinical examination showed a soft abdomen, with no rigidity and no pain. However, large masses in both lower quadrants of the abdomen were noted. No vaginal bleeding was observed, and the vaginal portion of the cervix appeared to be normal.

Ultrasound imaging showed two different abdominal masses > 15 cm (most likely belonging to a subserosal uterine leiomyoma and a left ovarian tumor), with no free fluid in the abdominal cavity. Blood tests analysis revealed general signs of inflammation, with a slightly increased white blood cell count and C-reactive proteins, as well as low hemoglobin levels. Other laboratory findings were normal, as shown in Table 1.

**Figure 1:**
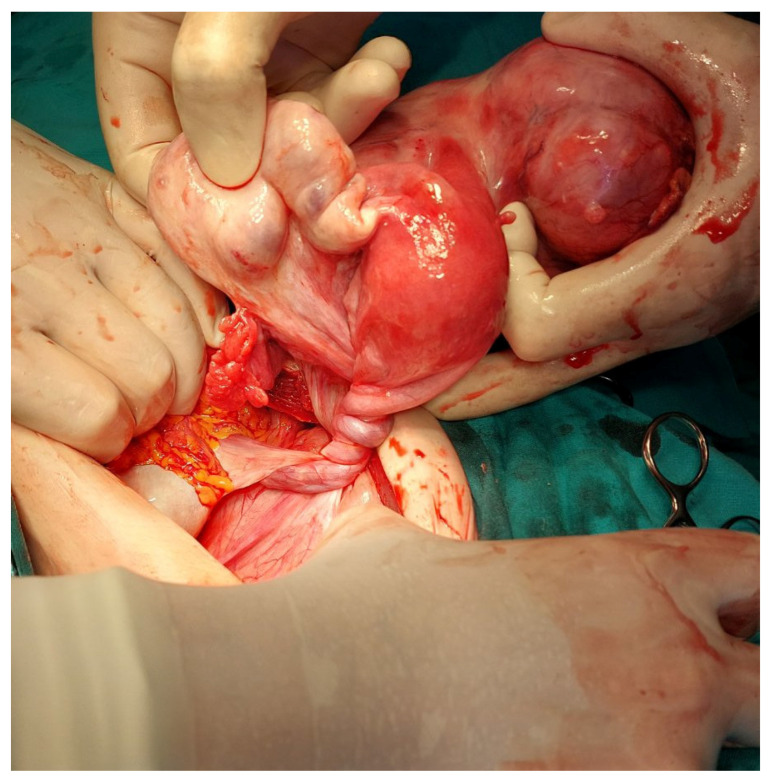
A 1080-degree torsion of the uterus, intraoperative.

A decision for an elective explorative laparotomy was made; however, one day after admission, an urgent surgical intervention was performed due to acute abdominal pain. Prior to the surgery, uterine torsion and the amputation of the cervix were still unknown to us; therefore, we suspected a torsion of a pedunculated subserosal uterine leiomyoma or an ovarian tumor torsion. Even though a pedunculated subserosal uterine leiomyoma and a left ovarian tumor were identified, there were no signs of torsion. Both adnexa and the uterine body were elevated from their normal positions in the true pelvis. The uterine body appeared normal but was rotated 1080 degrees counterclockwise (Figure 1), with no signs of congestion or necrosis.

**Figure 2:**
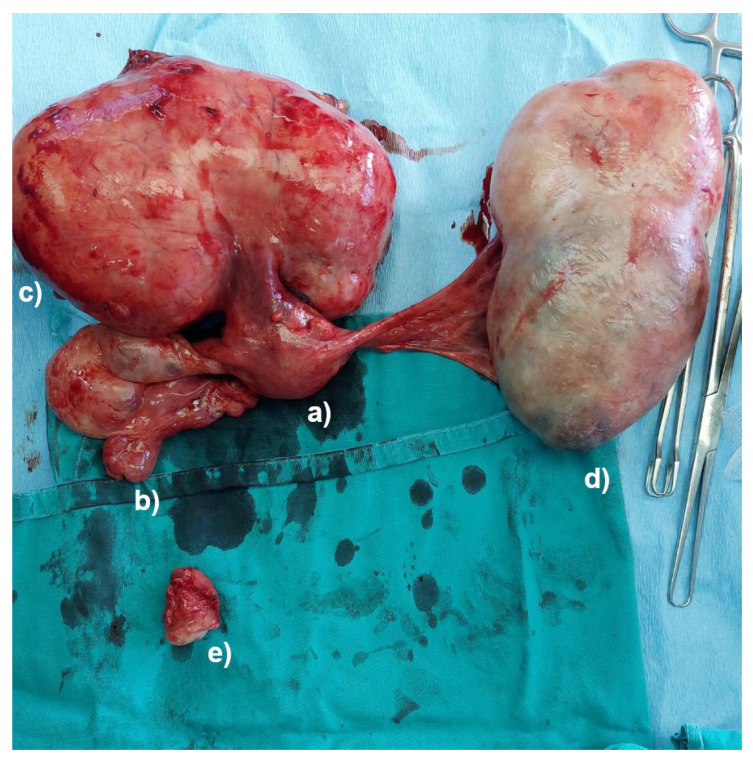
The specimen after surgery: the body of the uterus without the cervix (**a**), right adnexa (**b**), pedunculated leiomyoma (**c**), left ovarian tumor (**d**), and the amputated cervix (**e**).

**Figure 3:**
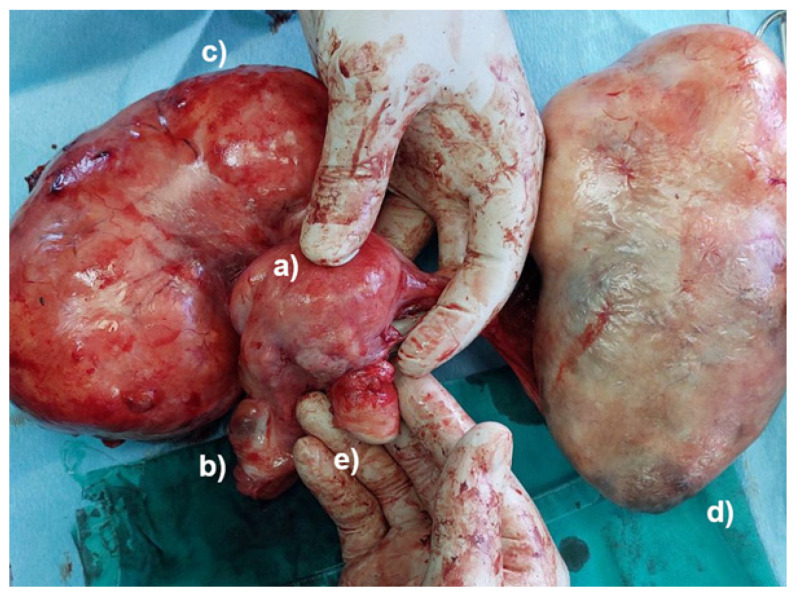
The specimen after surgery, connecting the separated parts: the body of the uterus without the cervix (**a**), right adnexa (**b**), pedunculated leiomyoma (**c**), left ovarian tumor (**d**), and the amputated cervix (**e**).

The lack of a uterine cervix was observed during the palpation of the junction between the uterine body and the uterine cervix. Complete abdominal hysterectomy and bilateral salpingo-oophorectomy were conducted, even though the vaginal portion of the cervix was missing. Further exploration of the abdominal cavity revealed an amputated vaginal part of the uterine cervix, with no signs of bleeding (Figure 2 and Figure 3).

A pathological examination of the specimen documented a uterine leiomyoma, accompanied by a left ovarian cystadenoma and an amputated vaginal part of the uterine cervix. Following the end of the surgical procedure, the patient remained stable, and her postoperative course was uneventful. She was discharged on the eighth postoperative day. No complications were detected 2 months after the surgery.

## 3. Discussion

We present a case of uterine torsion with a spontaneous amputation of the cervix in a non-pregnant, non-menstruating woman with a uterine leiomyoma and an ovarian tumor. A literature search on the platforms Medline and PubMed was undertaken, with the use of the following key words: ‘uterine torsion postmenopausal’ and ‘uterine cervix amputation’.

While reviewing similar studies, we found that authors reported cases of uterine torsion in 14 postmenopausal women, and no cases of uterine torsion with cervical amputation were reported. (Table 2)

The cause of uterine torsion remains unknown, although there is a link with pre-existing uterine anomalies, fibroids, or ovarian tumors [14,27,28]. It has been suggested that uterine torsion etiologies may differ, depending on the age of the patient [8]. In line with that, ovarian tumors and possible abnormalities of the reproductive organs may induce uterine torsion in the younger population of (menstruating) women. On the other hand, in older women (postmenopausal), uterine leiomyomas are the main reason for uterine torsion development [17,26], which corresponds with our case report findings.

The majority of uterine torsions are without any pathognomonic symptoms. Often, there are signs of abdominal pain (with differing intensity) and, sometimes, severe bleeding, which may lead to hemorrhagic shock [8,28]. In some cases, uterine torsion could have no reported symptoms [14,28]. Typically, laboratory findings provide evidence of non-specific inflammation, along with blood test disorders [8,27]. These observations are partially in line with results obtained in our report (Table 1). Therefore, precise and specific biomarkers of uterine torsion are still unknown [1]. However, a sign which may serve as a potential link to uterine torsion is the “whirl sign” of the uterine cervix (“twisted cervix”) [8,19,24]. This sign cannot be observed with the use of ultrasonography; only computerizing tomography (CT) or magnetic resonance imaging (MRI) could be utilized for this purpose [8]. On the other hand, some reports show that increased blood levels of some enzymes (creatine phosphokinase (CPK) and lactate dehydrogenase (LDH)), along with the clinical presentation, could be potential biomarkers for uterine torsion [12]. Furthermore, it has been shown that the presence of a large leiomyoma (diameter 15 cm and more) presents one of the key factors for uterine torsion development, especially in postmenopausal and non-gravid women [19], which is in accordance with the results obtained in our case. Uterine ligaments are responsible for the placement of the uterus inside the pelvic cavity. However, the presence of a massive leiomyoma for a longer time period may result in the weakness of uterine ligaments and, together with the gradual decrease in collagen production due to ageing, may provide initial steps for uterine torsion. All these factors may result in the unusual stretching of pelvic ligaments, leading to uterine instability and a higher possibility for uterine torsion. Uterine torsion, especially an acute torsion, could lead to reduced blood flow to the organ [1]. Simultaneously, uterine torsion blocks venous blood return, resulting in increased pressure inside the uterine cavity.

Therefore, any delays in uterine torsion diagnosis may result in irreversible changes due to reduced blood flow and serious complications, such as infarction and necrosis [12,15,16]. However, since the total number of uterine torsion cases is relatively small, indicating the right time for a surgical approach (to prevent potential serious and irreversible complications) is not possible. Therefore, appropriate analysis with CT and MRI should always be provided to avoid delaying or neglecting the uterine torsion diagnosis.

In this current case, we supposed that the uterine torsion was caused by a pedunculated subserosal leiomyoma and/or by an ovarian tumor. Torsion of either a leiomyoma or an ovarian tumor could have been expected. Instead of that, we found a torsion of the body of the uterus, accompanied by a leiomyoma and an ovarian tumor, which was followed by an amputation of the cervix. Interestingly, there were no signs of bleeding or hematoma. A possible explanation for the cervical amputation without acute symptomatology probably lies in the slow evolution of the process. At the start, there was a semi-torsion that caused gradual thrombosis of the blood vessels, and over time, the axial torque caused an amputation of the vaginal part of the cervix without bleeding, hematoma, or signs of an acute abdomen. The lack of LDH elevation could be explained by the fact that the amputation happened some time before the admission to the hospital. When the blood sample was taken, the necrotic process had already subsided, thus resulting in normal LDH levels. Acute pain that prompted immediate surgery was probably caused by a semi-torsion of the pedunculated leiomyoma or a semi-torsion of the ovarian tumor.

Taking into account the degree of uterine torsion, there is a possibility to develop a gangrenous uterus [12,15,16]. However, since uterus torsion is an uncommon condition, the precise duration until irreversible changes occur has not been established yet. Keeping in mind that abdominal pain of varying intensities is usually the only symptom of uterine torsion, prompt diagnosis is crucial for implementing an adequate procedure as soon as possible, with the intention of avoiding unnecessary complications and poor patient outcomes. Additionally, the surgical approach in case of uterine torsion may differ, depending on the age of the patient. Namely, in postmenopausal women, if there are signs of uterine necrotic changes due to persistent uterine torsion, hysterectomy is recommended. On the other hand, in younger patients, where there is a possibility to retain fertility, detorsion of uterus is proposed [11,28].

In some reported cases, it has been noted that a CT scan and/or MRI may provide an easier diagnosis of uterine torsion [29]. Our patient was scheduled for an MRI scan; however, the onset of acute abdominal pain necessitated an urgent surgical procedure.

Several CT or MRI findings may indicate uterine torsion, including ischemia and resulting infarction of the detected pelvic mass [8], a specific (X-shaped) configuration of the upper vagina [30], the presence of gas inside the uterine cavity [31], and the position of the pelvic mass, which is continuously changing [26], as well as other findings, such as hemoperitoneum [2] and the aforementioned ‘whirl sign’ (in some papers, it is called ‘whorl’ or ‘whorled’) [8,19].

## 4. Conclusions

Uterine torsion is an uncommon condition, especially in non-gravid and postmenopausal women, is difficult to diagnose, and can potentially result in serious complications. Despite the non-specific symptoms and non-specific laboratory data, uterine torsion should be considered an urgent condition with serious complications and outcomes.

To the best of our knowledge, this is the first reported case of a 1080° counterclockwise uterine torsion and an amputated cervix in a postmenopausal woman, together with uterine leiomyoma and an ovarian tumor. A CT scan and an MRI could be helpful in diagnosis, especially for finding the “whirl sign” of the uterine cervix. Early diagnostic procedures and prompt surgical intervention may provide better outcomes for women and prevent future complications.

## Figures and Tables

**Table 1:** Laboratory findings.

Elements	Values	Reference Values
White blood cells	13.3 × 10^9^ L	4.0–9.0 × 10^9^ L
Hemoglobin	103 g/L	110–170 g/L
Hematocrit	0.347 L/L	0.360–0.470 L/L
C-reactive protein	8.9 mg/L	0.0–5.0 mg/L
LDH	304 U/L	220–450 U/L
CPK	124 U/L	24–170 U/L

**Table 2:** Summary of the reported cases of uterine torsion in postmenopausal women (PubMed available articles from 2014 to 2024).

Authors	Age	Symptoms	Torsion Degree (°)
Halassy et al. [2]	70	Abdominal pain	180
Havaldar et al. [6]	55	Abdominal pain	180
Matsumoto et al. [8]	83	Abdominal pain in lower abdomen	90
Sikora-Szczęśniak et al. [11]	67	Abdominal pain	180
Oda et al. [12]	73	Abdominal pain	540
Yap et al. [15]	57	Abdominal pain in lower abdomen	180
Nagose et al. [16]	57	Abdominal pain	270
Chua et al. [17]	73	Abdominal pain in lower abdomen	NA
Wang et al. [18]	86	Abdominal pain in lower abdomen	360
Cheong et al. [19]	52	Abdominal pain in lower abdomen	720
Hasimoto et al. [20]	54	Abdominal pain in lower abdomen	180
Qin et al. [21]	83	Abdominal pain, nausea, vomiting	270
Ye et al. [22]	66	Abdominal pain in lower abdomen	540
Norström et al. [23]	68	Abdominal pain, vaginal bleeding	NA
Luk Y et al. [24]	61	Abdominal pain in lower abdomen	720
Matsumoto et al. [25]	73	Abdominal pain in lower abdomen	360
Jeong et al. [26]	87	Abdominal pain, fever	360

NA, not available.

## Data Availability

The data that support the findings of this study are available from the corresponding author upon reasonable request.
